# Fabrication of Phytic Acid/Urea Co-Modified Bamboo Biochar and Its Application as Green Flame Retardant for Polylactic Acid Resins

**DOI:** 10.3390/polym15020360

**Published:** 2023-01-10

**Authors:** Jinhuan Zhong, Enfu Wang, Yi Sun, Ningning Yin, Shuo Tian, Weijun Ying, Wenzhu Li, Wenbiao Zhang

**Affiliations:** 1 College of Chemistry and Materials Engineering, Zhejiang Agriculture and Forestry University, Hangzhou 311300, China; 2 Jiyang College, Zhejiang Agriculture and Forestry University, Shaoxing 311800, China

**Keywords:** bamboo biochar, oxidation, co-modification, flame retardancy, polylactic acid(PLA)

## Abstract

It is of great significance to develop green, sustainable additives to improve the thermal stability and flame retardancy of biopolymers. In this work, a synergistic modification of P/N elements to bamboo biochar (mBC) was successfully achieved by grafting a reaction of phytic acid and urea with preoxidized bamboo biochar. Fourier transform infrared spectroscopy, X-ray diffraction, nuclear magnetic resonance and scanning electron microscope determinations of the mBC demonstrated a successive grafting of phytic acid and urea to the originally porous surface. The ground mBC was blended with polylactic acid (PLA) to prepare mBC/PLA composites by extrusion and hot pressing. Mechanical strength studies showed a compromise in rigidity, which might originate from the mBC overdose and its limited miscibility with the resin. The thermogravimetric results supported the fact that the enhancement of thermal stability and flame retardancy of the composites with the mBC dosage, which showed that the mBC dosage in the PLA composites was not only lower than that of the conventional flame retardants, but also outperformed the counterparts using BC modified by inorganic phosphoric acid and urea. The mBC was prone to accelerate the earlier decomposition of the composites (30 °C lower in decomposition) and generate a continuous, dense residual carbon layer, which provides an effective shield resisting the mass and heat transfer between the combustion area and the underlying composite matrix. Only 10 wt% of mBC dosage could achieve a V-0 rating (UL94) for the composite, with a higher limiting oxygen index up to 28.3% compared to 20.7% for that of the virgin PLA; the cone colorimetric results also suggested that the flame retardancy had been greatly improved for all composites. In this work, biobased P-/N-containing bamboo biochar would be expected as a nontoxic biochar-based flame retardant that serves as green filler in polymer composites.

## 1. Introduction

Conventional petroleum-based polymers are commonly used in many applications for their good chemical and physical properties and cost-effectiveness, while their resistance to degradation has brought out quite a lot of environmental concerns [1,2], so there has been a boom in renewable polymers, particularly biodegradable biopolymers [3,4]. Nature source-deprived polylactic acid (PLA) is one of the most promising biopolymers, having attracted immense interest for its superb biocompatibility, biodegradability and raw material abundance [5], which makes it a versatile material in producing articles of consumption in medicine and hygiene, packaging, additive manufacturing and other industrial applications [6,7,8,9,10]. Nonetheless, the disadvantages of brittleness, low thermal stability and being ready to degrade at high temperature greatly hinder its potential applications in high-end use and other various commercial consumables and accessories. A great many studies have been reported on PLA modification to enhance its performance, such as molecular chain grafting [11], blending with other polymers [12,13,14], or modification with additives [15] to facilitate their processibility to achieve a better resistance.

Vast traditional additives and novel nanomaterials have been formulated with PLA resins to improve the flame retardancy [16,17,18,19,20], such as hydroxides, inorganic and organic phosphate and other hybrid chemicals; however, the loading amount of traditional additives is usually high, while the preparation of nanomaterials generally demands complicated maneuvers, which in return unintentionally limits its utilization in biopolymer formulation. Moreover, those flame retardants are either mineral resource-based or not easily degradable, which can cause some certain ecological issues. Biomass-based flame retardants may well be a candidate green and sustainable strategy to improve the thermal performance of PLA composites.

Carbonaceous materials [21], particularly nanomaterials such as graphene and carbon nanotube, are good candidates for a matrix structure to be modified with phosphors or nitrion elements to obtain intumescent flame retardants. Biochar could be a cost-effective consideration for chemical modification or grafting. Bamboo biochar or charcoal (BC) is a porous material produced by pyrolysis from the bamboo plant (and its processing residue), drawing great industrial interest for its distinctive structure and absorption properties [22].

Many studies have also been reported to show its favorable functions in polymer composites. You et al. [23] reported highly filled (up to 80%) bamboo charcoal powder-reinforced ultrahigh-molecular-weight polyethylene; Ho et al. [24] improved the mechanical, thermal and optical properties of PLA biocomposites using bamboo biochar. Zou et al. [25] investigated the effect of additional amounts of bamboo charcoal on mechanical and combustion properties of biodegradable BC/PLA composites; Sheng et al. [26] prepared a high-toughness PLA/bamboo cellulose nanowhisker bionanocomposite strengthened with silylated ultrafine bamboo-char; better mechanical properties were also obtained through bamboo charcoal as reinforcing fillers for polypropylene composites [27] when compared to coconut shell charcoal. Other versatile BC-based composites [28,29,30,31] featured with absorptive, conductive, or electromagnetic interference are typically followed with interest by researchers; it seems that this commercially available biochar has a particular meaning in the applications of polymer composites.

Apart from that, numerous works have been published on the flame retardancy of PLA composites [32]. Phosphor-based chemicals [33,34] are one of the most universal nonhalogen flame retardants used in industry, and low-cost inorganic phosphate minerals often act as both flame retardant and filler in polymeric formulation, usually requiring a high amount; phosphoric compounds are also conventional materials for modified resin or other matrixes to synthesize organophosphate flame retardants [35]. In the authors’ previous studies [36,37], aluminum hypophosphite (AHP) had been introduced into BC/PLA formulations, and the study suggested that the LOI of the BC/PLA(25/75) and BC/PLA/AHP(25/75/15) composites increased to 23.8 vol.% and 27.1 vol.%, respectively, as compared to 22.0 vol.% of virgin PLA, and their UL-94 test rated a V-2 and V-0 level, respectively.

In particular, green flame retardant has gained extensive attention for its sustainability. Xu et al. [38] synthesized a novel biobased flame retardant in aqueous phase (PA-AR) for PLA, with only 1 wt% addition of PA-AR making the PLA composite self-extinguish and enhancing the LOI value. A bio-based, core–shell flame retardant (APP@CS@ATMP) is designed to achieve excellent flame-retardant and antidripping properties of PLA composites [39]. Zhang et al. [40] employed a microwave-assisted modification method to prepare phosphorous acid/urea-based intumescent bamboo charcoal (BCm) that asserted the natural flame retardancy enhancement of BC/PLA composites; its LOI was 28.0 vol.% when 10 wt% of BCm was added, and 32.1 vol.% for 30 wt% addition, which was much greater than that of the 22.5 vol.% for BC/PLA(30/70) composite. Adding BC-m at 20 wt% or more gave a UL-94 rating of V-0 with significantly reduced melt dripping; meanwhile, its mechanical property was undermined to a certain extent but still was acceptable.

Stimulated by the concept of natural filler, phytic acid (inositol hexaphosphate, IP6) is frequently used for grafting or modification [41,42]. It can be regarded as an alternative source to prepare phosphate-based flame retardants because the bioderived IP6 offers multiple phosphonate groups (or phosphohydroxy) as reactive positions for grafting; it has been proven that an improved flame retardancy can be achieved by IP6 modification for organic coatings, fabric and polymers [43,44,45].

**Figure 1:**
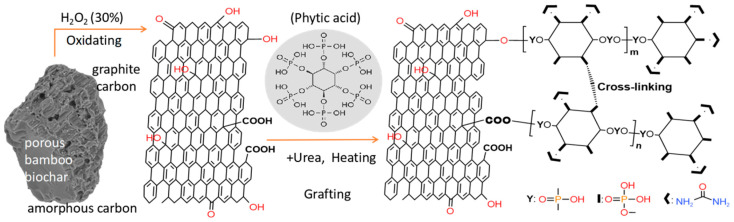
Schematic diagram of mBC fabrication.

In this work, a facile route is harnessed to fabricate phytic acid/urea co-modified bamboo biochar, that is, oxidized bamboo biochar (oBC) is firstly prepared in oxidant solutions, such as hydrogen peroxide or nitric acid; the oxygen-containing functional groups’ (e.g., -OH, -COOH and -C=O) abundance of oBC can thus provide themselves with reactive capability for the condensation reaction to IP6, whose spare phosphohydroxy groups can proceed with the reaction with urea or other amino compounds; the schematic diagram of fabrication can be briefly concluded in Figure 1. Hence, synergistic P-/N-co-modified bamboo biochar (mBC) can be finally fabricated by designating the biochar as flame-retardant matrix, and its flame-resistance investigations also indicate that the thermally-stable mBC is a biobased “three sources in one” intumescent flame retardant that can be applied as a green, efficacious flame-retardant filler for PLA composites.

## 2. Material and Methods

### 2.1. Materials

PLA resins (4032D, Cargill Dow, Nature Works Co. Ltd., Blair, NE, USA), powdered bamboo charcoal (300 mesh, carbonized at 650–750 °C for 72–96 h in limited oxygen condition in a brick kiln, Zhejiang Lvyi Bamboo Charcoal Ltd., Zhenjiang, China), hydrogen peroxide solution (30%, AR, Sinopharm Chemical Reagent Co., Ltd., Shanghai, China), phytic acid (50 wt%, AR, Sinopharm), urea (99 wt%, AR, Sinopharm) and ethanol (99 wt%, AR, Sinopharm) were all applied as received without any further purification.

### 2.2. Sample Preparation

#### 2.2.1. Synthesis of Phytic Acid/Urea Co-Modified BC (mBC)

Bamboo biochar (BC) was ultrasonically rinsed with ethanol water solution 3 times to thoroughly eliminate possible residue bamboo tar, ashes and dusts prior to oxidation, then hydrogen peroxide solution was gradually added (30%) to a flask, in which the dried BC could be placed in advance. The flask should be bathed in icy water to avoid the overspeed decomposition of hydrogen peroxide due to exothermic oxidation. The oxidized BC (oBC) can be obtained by filtration and drying or simply by evaporation of the suspension, which may take a longer time. The as-obtained oBC was mixed with phytic acid (IP6) at a mass ratio of 1:2 in a three-neck flask. The mixture was heated in several stages: firstly stirred at 75 ± 5 °C for 30 min, then at 100 ± 5 °C for another 30 min, proceeded by a reaction by adding urea solution to the mixture and making the IP6/urea mass ratio 1/1; the mixture continued to be heated to 120 ± 5 °C for 30 min and ultimately at 160 ± 5 °C until foaming. After these procedures, the foam was oven-dried at 110 ± 5 °C to prepare phytic acid/urea co-modified biochar (mBC), and later pulverized to powder form (200–325 mesh) and further rinsed with warm ethanol for at least 3 times to thoroughly remove possible residue chemicals. The standby mBC was dried to mix with PLA resins to prepare composites.

#### 2.2.2. Preparation of PLA Composites

**Figure 2:**
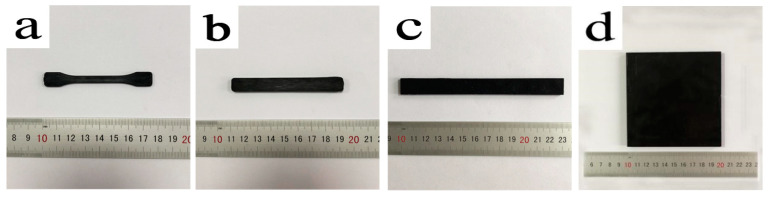
Sample images of PLA composites for measurements: tensile (**a**); flexural (**b**); LOI and UL-94 test (**c**) and CONE test (**d**).

Predried PLA resins and powdered mBC were used to prepare masterbatches using a traditional twin-screw extruder with the screw rotation speed at 15–20 rpm and temperature at 170–175 °C. The visually good masterbatches were used to fabricate specific composite samples by heat pressing at 175–180 °C. The formulations of the composites are shown in Table 1; a small amount (approx. 0.5–1.0%) of lubricant was used for easy processing. Virgin PLA samples were also fabricated as a reference. All samples are shown in Figure 2.

### 2.3. Measurements and Characterizations

#### 2.3.1. mBC and Residue Carbon Characterization

Fourier transform infrared (FTIR) spectroscopy of samples was measured with a spectrophotometer (Nicolet 6700, WI, USA) with a KBr slice to determine the changes of functional groups. The wavenumber ranged from 4000 to 400 cm^−1^.

The surface morphologies of mBC and cone-tested residue carbon of composites were observed by scanning electron microscopy (SEM, TM3030, Hitachi, Japan) with an accelerating voltage of 15 kV. Energy dispersion analysis X-ray system (Octane plus, Ametek, CA, USA) was used to analyze the elements of BCs and residue carbon samples recovered from the cone test. Samples were sputtered with gold for 90 s at a current of 15 mA prior to the observations.

X-ray diffraction (XRD) patterns were recorded by diffractometer (XRD-6000, Shimadzu, Japan) using Cu *Kα* radiation (λ = 1.542 Å) with a scanning step length of 2°/min at a range of 10°–80°. All samples were detected at a tube voltage of 40 kV and a tube current of 10 mA.

Carbon-13 nuclear magnetic resonance (^13^C-NMR) data were collected on a solid-state NMR spectrometer (Avance III HD 400, Bruker, Germany) at a spin rate of 10 kHz with magic angle spinning (MAS) using a 4 mm rotor. The resonance frequency was set to 400 MHz. All samples for analyses were gently ground to ensure homogeneity. All spectra were acquired as the result of 1024 scans, and the prescan was delayed by 6.5 μs.

X-ray photoelectron spectroscopy (XPS) was employed to investigate the element contents of BCs and the cone-tested samples, which was completed using a Thermo Scientific K-Alpha spectrometer (Thermo Fisher Scientific Inc., Waltham, MA, USA) equipped with a monochromatic Al Kα X-ray source (1486.6 eV) operating at 100 W. The samples were analyzed under vacuum (P < 10^−8^ mbar) with a pass energy of 150 eV (survey scans) or 25 eV (high-resolution scans). All peaks were calibrated with C 1s peak binding energy at 284.8 eV for adventitious carbon. The experimental peaks were fitted using Avantage software (version 5.1).

#### 2.3.2. Mechanical Properties

The mechanical properties of composites were evaluated by using a microcomputer-controlled electronic universal testing machine (CMT610, MTS Industrial Systems, Shenzhen, China) in accordance with industrial standards GB/T 1040 (tensile strength) and GB/T 9341 (flexural strength), respectively. For tensile testing, the width and thickness of the narrow stress zone in the samples were 5 mm and 2 mm, respectively. The gauge length was set at 60 mm and the speed at 2 mm/min. The sample dimensions for flexural testing were 80 × 10 × 4 mm, and the loading span was set at 60 mm and speed at 2 mm/min.

#### 2.3.3. Thermal Stability

Thermogravimetric analysis (TGA) was carried out using a thermogravimetric analyzer (TG 209F3 Tarsus, Netzsch, Germany) under a nitrogen atmosphere. Approximately 5–10 mg of samples was placed in an aluminum crucible, run under a nitrogen flow rate of 20 mL/min and heated at a range of ambient temperatures to 800 °C with heating rate at 10 °C/min. A blank crucible was used as a reference.

#### 2.3.4. Combustion Properties

The limiting oxygen index (LOI) was conducted by oxygen index meter (JF-3, Jiangsu Jiangning Analysis Instruments Co., Ltd, Nanjing, China) which expressed as vol.% minimum concentration of oxygen required to initiate composite combustion in accordance with ISO 4589-2:2017. All sample dimensions were 100 × 10 × 4 mm.

The vertical flame test was investigated by a Type 5402 vertical combustion tester (Suzhou Yangyi Woerqi Detection Technology Co., Ltd., Jiangsu, China) in accordance with ASTM D3801. All sample dimension was 130 × 13 × 3 mm_._

The combustion test was determined with a cone calorimeter (CONE, FTT007, Fire Testing Technology, West Sussex, UK) in accordance with ISO 5660-1, All sample dimensions were 100 × 100 × 4 mm. Sample sitting horizontally in a bed of aluminum foil was located in combustion chamber with a predetermined test height at 25 cm, then ignited, and all combustion results were recorded until the combustion was completed with an external heat flux of 35 kW/m^2^.

## 3. Results and Discussion

### 3.1. Characterization of BC, oBC and mBC

**Figure 3:**
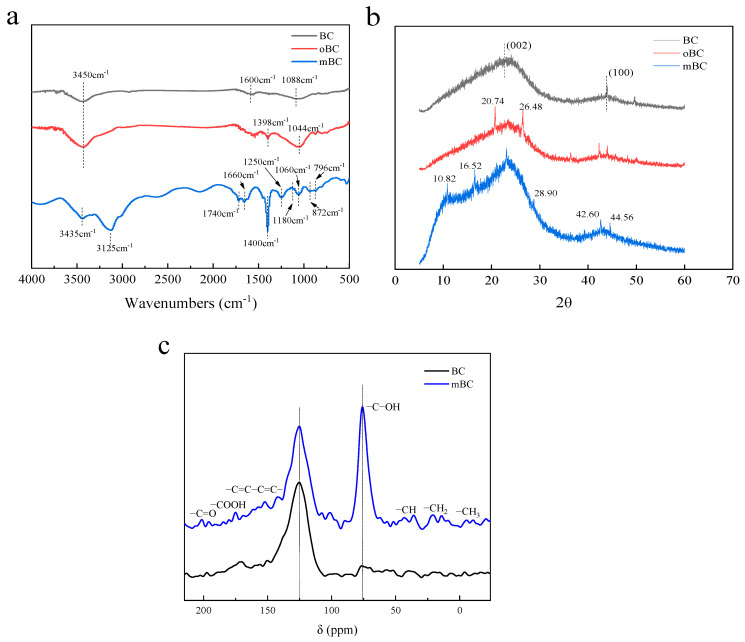
FTIR (**a**), XRD (**b**) and ^13^C-NMR (**c**) spectra of BC, oBC and mBC.

The FTIR spectra depicted in Figure 3a were applied to study the differences between BC, oBC and mBC. The as-received BC did not present any obvious diffraction peaks except for evidence of an amorphous carbon structure of nature (biochar) at wave numbers of 3450 cm^−1^ (–OH), 1600 cm^−1^ (C=C) and 1088 cm^−1^ (C–O), respectively; while for oBC, the appearance of peaks at 3450, 1690 and 1044 cm^−1^ (C=O) were immensely intensified, which corresponded to the introduction of carboxylic and phenolic (hydroxyl) groups at the BC surface, therefore making the successive grafting possible. There was an obvious contrast between the thoroughly rinsed mBC with the former two samples; as seen in Figure 3a, the peak at 1250 cm^−1^ was attributed to the –P=O bond that stemmed from the reaction of phosphohydroxy (from IP6) to the oxygen-containing groups (carboxylic or hydroxyl) of the oBC surface, and the remaining adsorptions at 1400, 1180, 1060, 872, and 796 cm^−1^ were attributed to C–N, C–N. The symmetrical stretching of P–O and the asymmetrical stretching of the P–O and P–O–P bonds, respectively, occurred for the same reason; it was also easy to distinguish the characteristic peaks distributed at 3125 cm^−1^ (N–H) owing to the synergistic grafting of urea to spare phosphohydroxy groups. The absorption peak at 1740 cm^−1^ may be attributed to O-C=O, and 1660 cm^−1^ to C=C. The emergence of these characteristic peaks illuminated the successful synthesis of organophosphate- and nitrogen-co-modified bamboo biochar.

XRD data were analyzed to further confirm the differences between virgin BC and mBC. Virgin BC did not claim any clear and definite diffraction peaks, as shown in Figure 3b, merely representing the amorphous structure of carbon (biochar). The broad peaks at 2θ = 20°–30° generally denoted carbon reflection from the stacked hexagonally structured graphite (002), and the weak peak around 2θ = 43° revealed that pyrolytic carbon was derived from the hexagonal structure (100).

Unlike virgin BC, the oxide BC gained obvious peaks at 2θ = 20.74° and 26.48°, and mBC had a good deal of crystal structures, for instance, peaks at 2θ = 10.82°, 16.52°, 28.90°, 42.60°, and 44.56°, respectively, which undoubtedly disclosed that the as-fabricated mBC had produced new crystal structures through the coordinative modification of IP6 and urea; these structures were homologous to the traditional polyphosphate-based flame retardants, which could be deemed solid proof of the grafting to the BC surface.

The structural differences of virgin BC and mBC detected by ^13^C-NMR were shown in Figure 3c. Bamboo biochar was quite complicated in graphite and amorphous carbon structures. The spectra for biochar mainly consisted of the band for aromatic (graphite) carbons around 125 ppm, featured by the changing trend of ^13^C spectrum at high temperatures; small resonance peaks of C=O (210–180 ppm), O=C–O (185–165 ppm), C=C–C=C (160–140 ppm), C–OH (85–45 ppm), and alkyl groups (60–10 ppm) were seen from the NMR spectrum. After modification, a strong C–OH appeared and the remaining small peaks moved to the high field or almost vanished, indicating that through the formation of new bonds during the grafting, the usual order of 13C may be rearranged. It provided a confirmation of IP6/urea interacted with biochar during the mBC fabrication.

**Figure 4:**
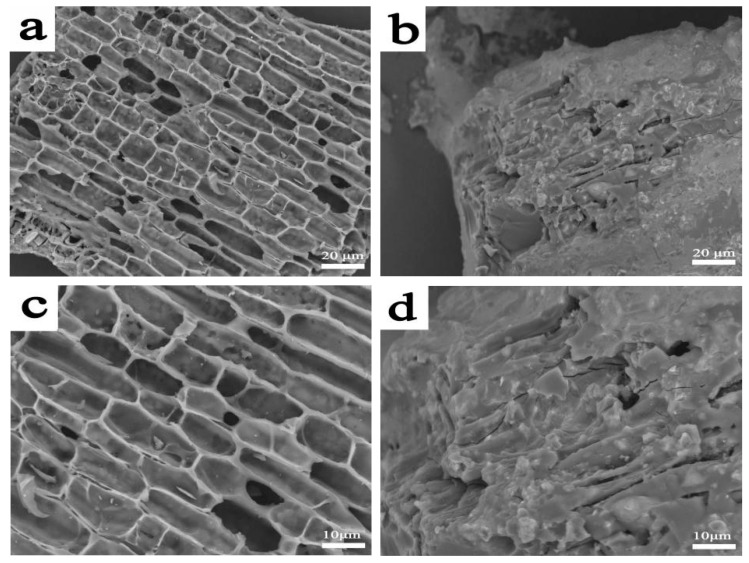
Topography of BC (**a**,**c**) and mBC (**b**,**d**) with different magnifications.

The surface topographies of BC (a,c) and mBC (b,d) observed by SEM are seen in Figure 4, from which it can be noticed that amorphous BCs presented with porous structures (Figure 4a) that developed from original bamboo parenchyma tissues; the porosity can afford an adequate specific surface area that includes liberally active spots for the synergistic grafting of phytic acid (IP6) and urea molecules to the BC substrate. After the co-modification, the classical morphology vanished on the whole, and the pores were too obscure to be observed any more; the IP6 and urea were likely to suffuse across the porous substrate. The primitive hierarchical structure of BC that could help to anchor to the resins was leveled off to devastate the interlocking occurrence between resins and the fillers, which could be an induction to detriment of the mechanical properties of the composites in applications.

### 3.2. Mechanical Properties

**Figure 5:**
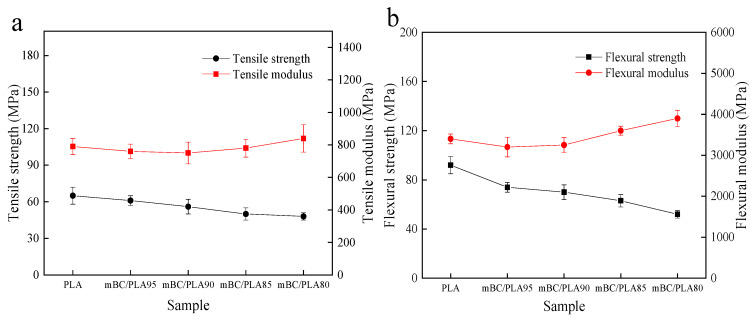
Tensile (**a**) and flexural (**b**) properties of PLA composites (error bars represent standard deviation).

The tensile and flexural performance of virgin PLA and composites are shown in Figure 5, with the average values and standard deviations listed in Table 2. Tensile and flexural results of composites was quite a contrast to those of virgin PLA resins. A notable reduction in strength could be identified for all samples when compared to virgin PLA. The rigidity of the composites were prone to decline with a gradual dosage increase in mBC to the PLA resins, that is, both a tensile strength drop from 61.54 MPa (mBC/PLA95) to 48.01 MPa (mBC/PLA80) and a flexural strength drop from 74.13 MPa (mBC/PLA95) to 52.45 MPa (mBC/PLA80), whereas the deformation resistance enhanced, that is, the flexural modulus was slightly increased with the mBC dosage in composites; on the other side, their tensile modulus fluctuated and basically changed alike, and the same tendency occurred for the BC/PLA control groups [36].

In addition, the mBC in the PLA matrix was incidental to deducing aggregation or local accumulation to the composites, which in return contributed to the reduction. The surface-modified biochar had slightly smooth substrate (see in Figure 4b,d); it might not have exerted a filler effect in the PLA matrix, causing a deficient interaction of mBC particles with each other in the matrix that was incapable of enhancing the toughness effectively, while it also jeopardized the stress transmitting during the measurement, and it came up with the inevitable consequence of a strength reduction in the PLA composites when compared to the control groups. For that matter, the reduction was presumably caused by the possible incompatibilities of the inert PLA resins to the surface-active modified biochar particles that were rich in phosphor/nitro-based groups. Although mechanical property was compromised to a certain extent with the additive addition, it still was acceptable (actually higher) according to the technical requirements for industrial purpose.

### 3.3. Thermal Stability

**Figure 6:**
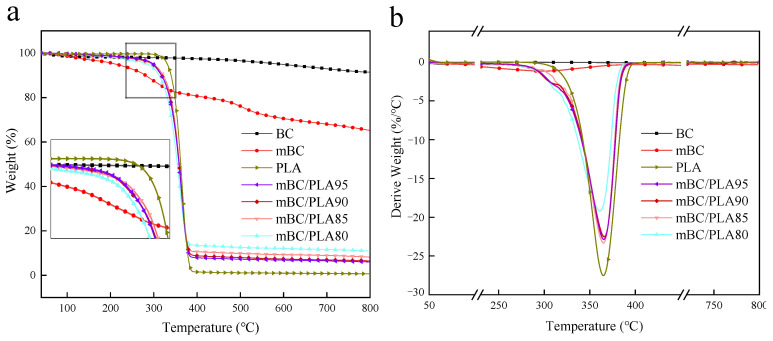
Thermogravimetry (**a**) and derivative thermogravimetric (**b**) curves for virgin BC, mBC and mBC/PLA composites analyzed under nitrogen atmosphere.

The thermogravimetry (TGA) and derivative thermogravimetric (DTG) results of virgin BC and mBC are shown in Figure 6. It is quite straightforward to see through the pyrolysis performance among BC and mBC that the former saw a fairly trifling mass loss at the terminal of the pyrolysis, and there was briefly no significant mass variance of samples in the whole range, and the carbon residue rate just gradually dropped and finally concluded with a relatively high level (91.4%), even when the pyrolysis ended at 800 °C; however, the latter gave out a steep mass loss at 298 °C with a mass drop of approx. 12.22% in total.

The pyrolysis of mBC at a lower temperature (lower than that of PLA) would be expected to simultaneously release acidic phosphates and inflammable ammonia, stimulating the earlier onset of the degradation of PLA composites, thus restraining the fire because of the breakdown of the “three sources (carbon, acid and gas) in one” flame retardant during combustion.

Thermogravimetric analysis data of PLA composites in Table 3. The degradation onset temperature (T_5%_, temperature at which the sample undergoes a 5% mass loss) and maximum temperature of thermal degradation rate (T_max_) of the samples were clearly differed. In Table 3, it was also exhibited that both the T_5%_ and T_max_ data of the mBC/PLA composites were 30 °C lower than those of the virgin PLA resins, who were ready to quickly pyrolyze at 331 °C, as shown in Figure 6b and Table 3, and its T_max_ at 366 °C; most of the sample masses were lost at 400 °C or beyond, the corresponding carbon residue rate was merely 1.35% at 800 °C, and more leftover carbon had been remained, as seen in Table 3, indicating that the additive’s earlier degrading could help the combustion resistance of the composites. In addition, when mBC pyrolyzed at 400 °C and beyond, its mass variance stabilized with a 1/3 mass loss at the end of the test, which may well be attributed to the evaporation and dehydration of water, the emission of small molecular phosphor- or nitrogen-containing gases/compounds, the in-depth carbonization of BC substrate, and the thermal degradation of carbon residue in the process of heating up. It somehow confirmed that the gas phase and condensate phase of the flame retardant (mBC) were formed during combustion, which reasonably accounted for the carbon residue decrease as compared to that of the virgin BC counterpart.

### 3.4. Combustion Performance

#### 3.4.1. LOI and UL94 Measurements

**Figure 7:**
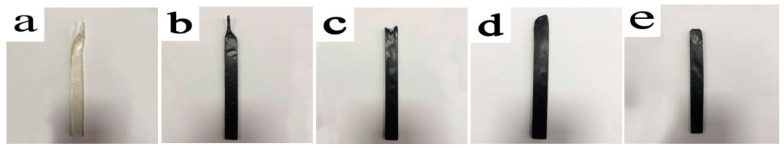
Sample images of PLA composites after vertical flame test: PLA (**a**); mBC/PLA95 (**b**); mBC/PLA90 (**c**); mBC/PLA85 (**d**); mBC/PLA80 (**e**).

Table 4 displays the limiting oxygen index (LOI) and UL-94 results of virgin PLA resin and composites. It was easy to ignite virgin PLA resins for whose LOI is approximately 20%, an equivalent amount to the oxygen level in air; apparent dripping could also be seen during the ignition, and no rating (NR) was available in the UL-94 test. Sample images of composites after the UL-94 test are shown in Figure 7. Nevertheless, almost all mBC/PLA composites gained a remarkable leap in fire resistance; the LOI value could approach as high as 30% for a 15% addition. Moreover, dripping could only be observed for the relatively lower addition (5 wt%) of mBC to the composite, and the cotton underneath did not catch fire; the remaining composites all achieved a V-0 level when mBC was dosed at 10 wt% or beyond, and the dipping was also constrained. The LOI and UL-94 results both outperformed the BC/PLA and BC-m/PLA composites [40]. With reference to the BC-m/PLA composites, they were reported to be prepared through phosphorous acid and urea with almost same modification conditions, except for microwave assistance. The organophosphonate acid (phytic acid, IP6)-modified BCs in this work seem to offer an improved flame resistance performance when compared to their inorganic phosphoric acid (PA) counterparts. The difference may originate from the more intensified networking on the surface of bamboo biochar constructed by grafting hexagonal-structured phytic acid that affords more reactive spots reacted with urea molecules; hence, the high modification degree perhaps rationalized the excellence of mBC/PLA composites in the fire resistance test, but on the other side, the linear-molecular phosphorous acid might not form a likewise crowded and thick grafting framework as its IP6 did; for this reason, the fire resistance tests had technically eventually supported the explanation in this work, and herein lies the potential that the biomass-based mBC could be applied as a green flame retardant for polymer formulations.

#### 3.4.2. Cone Measurement

**Figure 8:**
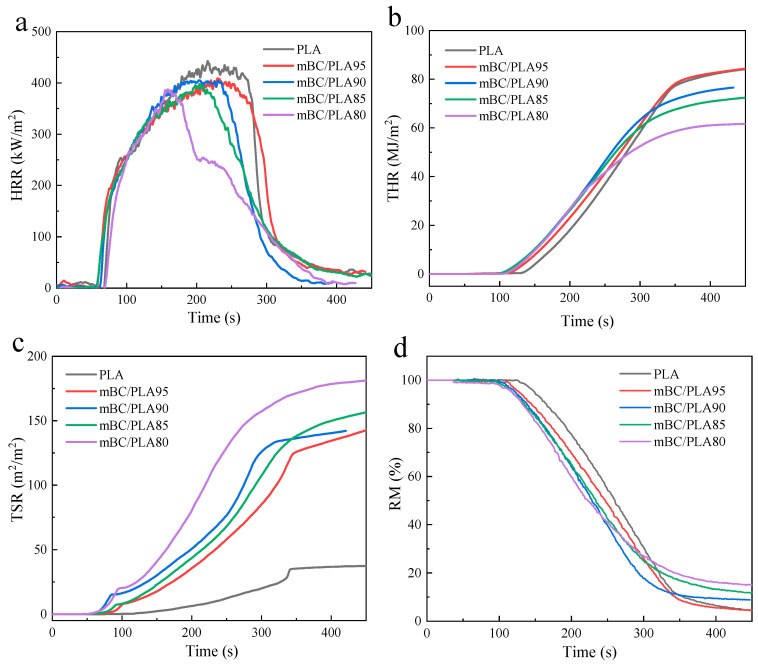
Cone analysis of PLA composites with an external heat flux of 35 kW/m^2^: HRR (**a**); THR (**b**); TSR (**c**); RM (**d**).

Cone calorimeter (CONE) tests were usually conducted as standard practical test to evaluate the flame resistance of materials. The main test results for the composites are listed in Table 5 and Figure 8. It was known that the virgin PLA resins had no notable flame retardancy, and this was evidenced by the test data in Table 5. All mBC/PLA composites surpassed the resin in each indicator in the cone test. The mBC dosage contributed an obvious shrinkage of time to ignite (TTI). The TTI for virgin PLA was approximately 63 s, and the TTI decreased to 51 s with a 5 wt% dosage of mBC to the resins, which would give rise to the formation of phosphate and ammonia gas that was released from the earlier decomposition of mBC, accelerating the dehydration and charring to form a useful carbon layer on the matrix; meanwhile, it would contribute to the resins’ degradation and postpone/constrain the fire diffusion. The TTI data implied that the more mBC dosed in the composites, the more effective and efficient it was in arriving the fire extinction.

Total heat release (THR) was also an important factor reflecting potential risk fire spreading. Still, from Table 5 it was easy to learn that the THR and peak HRR (pHRR) results for the mBC/PLA composites were much lower than those of the virgin PLA resin, whose THR and pHRR were 86.09 MJ/m^2^ and 447.26 kW/m^2^, respectively; they declined gradually with the growing mBC dosage, and ended up with 61.77 MJ/m^2^ and 389.56 kW/m^2^, respectively, when the dosage increased to 20%. Similar to the pHRR changes, the peak mass loss rate (pMLR) bumped for the mBC/PLA90 composite, but after that, a decreasing tendency could still be observed in the range of 0.56–0.59 g/s in contrast to 0.72 g/s that for the virgin resin, which clarified the effectiveness of the mBC for the PLA formulations. Total smoke release (TSR) seemed to take a great leap for the composites in the current study, which should be further improved in future work.

Moreover, it appeared that there was a remarkable increase in carbonaceous residues with the mBC dosage; the residual mass (RM) could be expected to be greater than 10% when the dosage went to 15% or more. Notably, the actual RMs of the mBC/PLA composites with a mBC dosage of 10–20 wt% were attained, with better results of 8.82%, 10.23% and 14.97% wt%, respectively, outperforming by 36.3%, 24.5% and 37.6% those of the theoretical carbon residue rate results (6.47%, 8.22% and 10.88%, as seen from the thermogravimetric data in Table 3), confirming the strengthened thermostability of the composites with mBC application. However, the slim dosage of mBC in the composites in this work produced fewer carbon leftovers than the theoretical result, which also demonstrated the incapability of forming an intact carbon layer protecting the PLA matrix as well.

### 3.5. Residual Carbon Analysis

**Figure 9:**
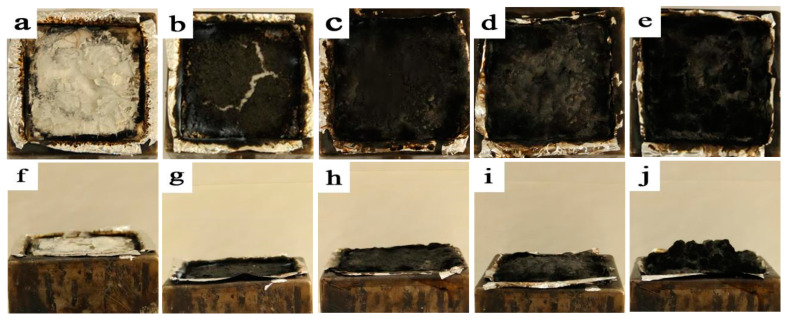
Top and oblique views of cone-tested composites: virgin PLA (**a**,**f**); mBC/PLA95 (**b**,**g**); mBC/PLA90 (**c**,**h**); mBC/PLA85 (**d**,**i**); and mBC/PLA80 (**e**,**j**).

The digital images of the CONE-tested virgin PLA and its composites were shown in Figure 9. The results (Figure 9a) showed that virgin PLA was virtually non-flame-resistant, taking an almost-complete burning with few leftovers. While the residual carbon could be seen without the aid of instruments for the remaining mBC/PLA composites, there was a visible rift across the residual carbon layer (Figure 9b) that abruptly sabotaged the physical intactness of the residual carbon layer covering the aluminum foil when the BCm dosed at 5 wt%; since the mBC dosage increased to 10 wt% and above, perfectly plain and continuous coverings on the foil (Figure 9c–e) were seen, which indicated that a protective barrier had been built against the flame attack. It was also found by hand feeling that the residual carbon was all in puffy foam form, verifying that the biobased mBC was a concrete intumescent flame retardant; when the addition of the mBC increased to 20 wt%, the residue volume of the after-cone test took a sudden intumescence that outbound the supportive framework, in contrast to a slight expansion occurring in the mBC/PLA85 (mBC dosage at 15 wt%). The expansive residue could validly diminish the possibility of heat and mass transferring between the gas phase and the condensed phase during the combustion, which in return opted to constrain the fire spreading. Lastly, similar flame resistance performance (forming a continuous carbon layer) was gained for the BC-m/PLA composites [40] when the BC-m loading was 20 wt% or 30 wt%; therefore, it made sense from the outweighed results that the organophosphate-modified BC could be a potential candidate as a phosphor source in fabricating a green and nontoxic BC-based flame retardant in the future.

**Figure 10:**
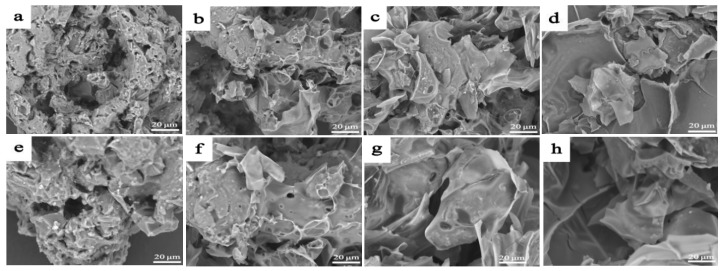
Microscopic morphology of residual carbon from cone-tested composites: mBC/PLA95 (**a**,**e**); mBC/PLA90 (**b**,**f**); mBC/PLA85 (**c**,**g**); and mBC/PLA80 (**d**,**h**).

The microscopic morphology of the residual carbon was further investigated by scanning electron microscopy (SEM) and is displayed in Figure 10, from which it can be observed that the structure of the residue most likely resembled it, but differed in details. There were plenty of holes and voids pervading the surface of the residue recovered from mBC/PLA95, which could possibly make it an Achilles’ heel in confronting fire invasion; they could be regarded as versatile channels for heat dispersal and volatile release, and the latter could effectively undermine the flame suppression. By contrast, the mBC/PLA90 composite could give a sound carbon layer with fewer defects, providing a solid firewall that limits the combustion. When more mBC was added to the PLA resins, the corresponding structure of the residue was reinforced with a better shielding performance, defending the substrate underneath in fire, as proven by the discussed results.

**Figure 11:**
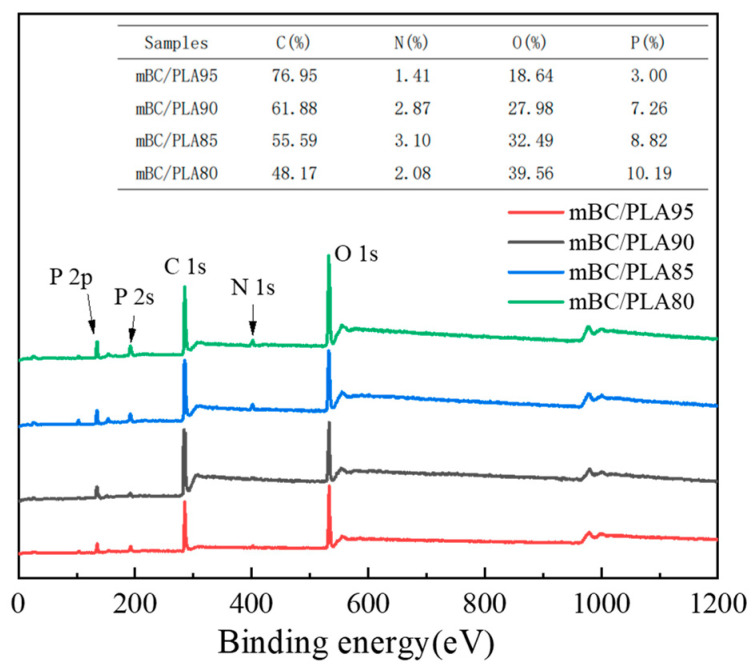
XPS results of residual carbon from cone-tested composites.

The chemical components of the char residue after CONE testing were determined by XPS method, and the spectra are expressed in Figure 11. All residues mainly consisted of the elements C and O with a total amount of 88% or above, besides mBC/PLA, whose P/N content attained less than 5%, which accounted for its relatively weak flame resistance, yet it was up to 12% for the remaining composites, as seen from the insert in Figure 11, which played an important role in ensuring the P/N synergistical effect on the biobased flame retardant.

## 4. Conclusions

Phytic acid/urea co-modified bamboo biochar (mBC) was successfully fabricated and applied as a green flame retardant for polylactic acid (PLA). A remarkable increase in phosphor and nitrogen content had been demonstrated by the characterization of FTIR, XRD, ^13^C-NMR and SEM on the mBC samples in contrast to bamboo biochar, which proved that the organophosphate acid and urea had been synergistically grafted to the biochar. The mechanical property assessment revealed an acceptable reduction in strength occurring in the mBC/PLA composites that may have resulted from the incompatibility of the P/N-modified biochar and the incompetent intervention of the resins to mBC. Flame retardancy studies found that the PLA composites had attained a satisfactory V-0 rating (UL94) for a 10 wt% dosage of mBC, and the LOI value could reach up to 30 vol.% when the dosage increased to 15 wt% or beyond. The CONE results expounded that the combustion performance would be greatly enhanced with lower THR and HRR and higher RM with regard to mBC dosing, which confirmed the mBC as a versatile flame-retardant filler in PLA composites. The thermal stability of the mBC was certainly guaranteed by the synergetic modification of phytic acid and urea on the BC substrate; it deduced the resins’ earlier onset of decomposition and carbonization that accelerated the protective residual carbon layer generation, thus containing the fire‘s rage. The microscopic observations of the residue also suggested that a dense, continuous structure was established to counteract the flame influence, which illuminated the excellence and superiority of this biobased flame retardant. Briefly the nontoxic BC-based flame retardant in this work can be served as green filler in polymer composites.

## Figures and Tables

**Table 1:** Formulations for mBC/PLA composites.

Sample	Ingredients (wt%)		
	PLA	mBC	Lubricant
Virgin PLA	100	0	0.5–1
mBC/PLA95	95	5	0.5–1
mBC/PLA90	90	10	0.5–1
mBC/PLA85	85	15	0.5–1
mBC/PLA80	80	20	0.5–1

**Table 2:** Mechanical properties of PLA composites ± stdev.

Sample	Tensile Strength (MPa)	Tensile Modulus (MPa)	Flexural Strength (MPa)	Flexural Modulus (GPa)
Virgin PLA	65.25 ± 7.21	790.65 ± 50.27	92.52 ± 7.21	3.40 ± 0.12
mBC/PLA95	61.54 ± 4.52	761.54 ± 45.11	74.13 ± 4.12	3.20 ± 0.24
mBC/PLA90	56.22 ± 6.10	750.84 ± 67.89	70.20 ± 6.32	3.25 ± 0.18
mBC/PLA85	50.35 ± 7.21	779.61 ± 55.74	63.19 ± 5.25	3.61 ± 0.11
mBC/PLA80	48.01 ± 3.02	840.66 ± 84.75	52.45 ± 3.02	3.90 ± 0.20

**Table 3:** Thermogravimetric analysis data of PLA composites.

Sample	T_5%_ (°C)	T_max_ (°C)	Carbon Residue Rate (wt%)		
			400 (°C)	600 (°C)	800 (°C)
BC	593	365	97.46	94.87	91.40
mBC	207	298	80.65	70.35	65.21
PLA	331	366	2.28	1.63	1.35
mBC/PLA95	299	337	7.84	6.82	5.98
mBC/PLA90	291	337	8.70	7.30	6.47
mBC/PLA85	296	338	10.60	9.25	8.22
mBC/PLA80	280	329	13.42	12.06	10.88

**Table 4:** LOI and UL-94 results.

Samples	LOI (%)	UL-94 Test	
		Dripping	Rating
Virgin PLA	20.3	Yes	NR
BC/PLA90 *	21.2	Yes	NR
BC/PLA80 *	21.8	Yes	NR
BC-m/PLA90 *	28.0	Yes	V-2
BC-m/PLA80 *	29.2	No	V-0
mBC/PLA95	24.7	Yes	V-2
mBC/PLA90	28.3	No	V-0
mBC/PLA85	29.9	No	V-0
mBC/PLA80	31.2	No	V-0

* Note: data from [40].

**Table 5:** Main CONE test results for mBC/PLA composites.

Sample	TTI (s)	THR (MJ/m^2^)	pHRR (kW/m^2^)	pMLR (g/s)	TSR (m^2^/m^2^)	RM (%)
Virgin PLA	63	86.09	447.26	0.72	36.84	2.98
mBC/PLA95	51	85.61	411.77	0.59	149.40	3.77
mBC/PLA90	43	76.58	425.96	0.76	142.19	8.82
mBC/PLA85	40	74.16	402.52	0.58	165.64	10.23
mBC/PLA80	38	61.77	389.56	0.56	181.56	14.97

Notes: TTI, time to ignition; THR, total heat release; pHRR, peak heat release rate; pMLR, peak mass loss rate; RM, residue mass rate.

## Data Availability

Data available on request from the authors.
